# Disappearance of a disconnected pancreatic duct after pancreatic duct decompression in a pediatric case of walled-off necrosis (with video)

**DOI:** 10.1097/eus.0000000000000181

**Published:** 2026-05-14

**Authors:** Masaki Miyazawa, Masahiro Yanagi, Hajime Takatori

**Affiliations:** Department of Gastroenterology, Kanazawa University Hospital, Kanazawa, Ishikawa, Japan.

A 13-year-old boy undergoing L-asparaginase therapy for acute lymphoblastic leukemia developed severe necrotizing pancreatitis that progressed to a large walled-off necrosis (WON) [Figure 1]. EUS-guided drainage using a lumen-apposing metal stent (Hot AXIOS; Boston Scientific, Marlborough, MA, USA) and multiple sessions of endoscopic necrosectomy were performed [Figure 2]; however, the necrotic collection failed to regress sufficiently despite repeat debridement [Figure 3A, B]. During necrosectomy, contrast injection into the WON demonstrated opacification of the main pancreatic duct in the pancreatic head, confirming disconnected pancreatic duct syndrome (DPDS) [Figure 3C and Video 1]. We considered that persistent leakage of pancreatic secretions into the WON due to DPDS contributed to ongoing inflammation and failure of the cavity to regress.

**Video 1.** 

**Figure 1.**
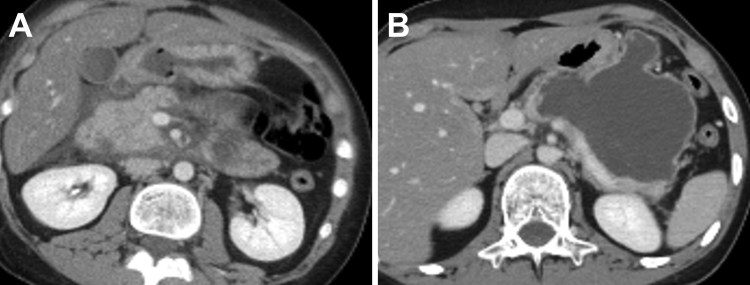
Evolution from acute necrotizing pancreatitis to walled-off necrosis (WON). (A) Contrast-enhanced CT at the onset of acute necrotizing pancreatitis. (B) Follow-up CT demonstrating the development of a large walled-off necrosis. CT, computed tomography.

**Figure 2.**
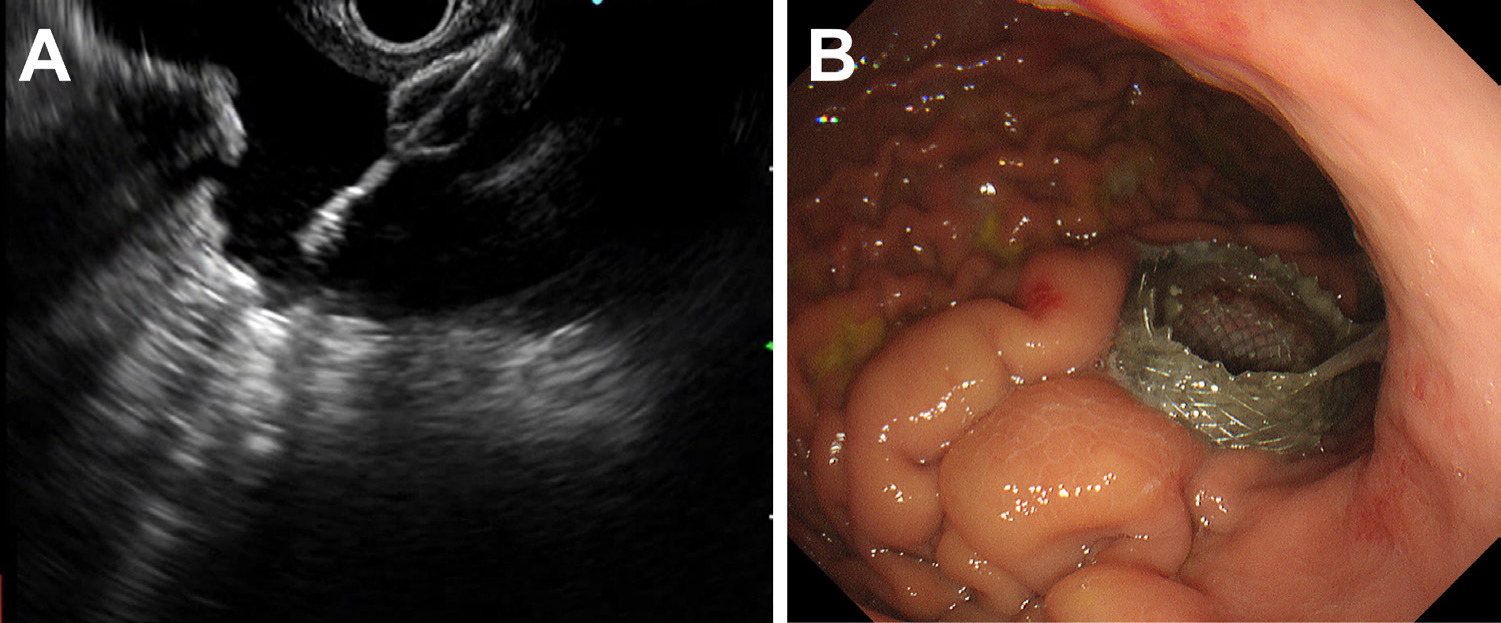
EUS-guided drainage of the WON using a LAMS. (A) EUS image showing needle puncture into the WON cavity. (B) Endoscopic image confirming successful LAMS deployment. LAMS, lumen-apposing metal stent; WON, walled-off necrosis.

**Figure 3.**
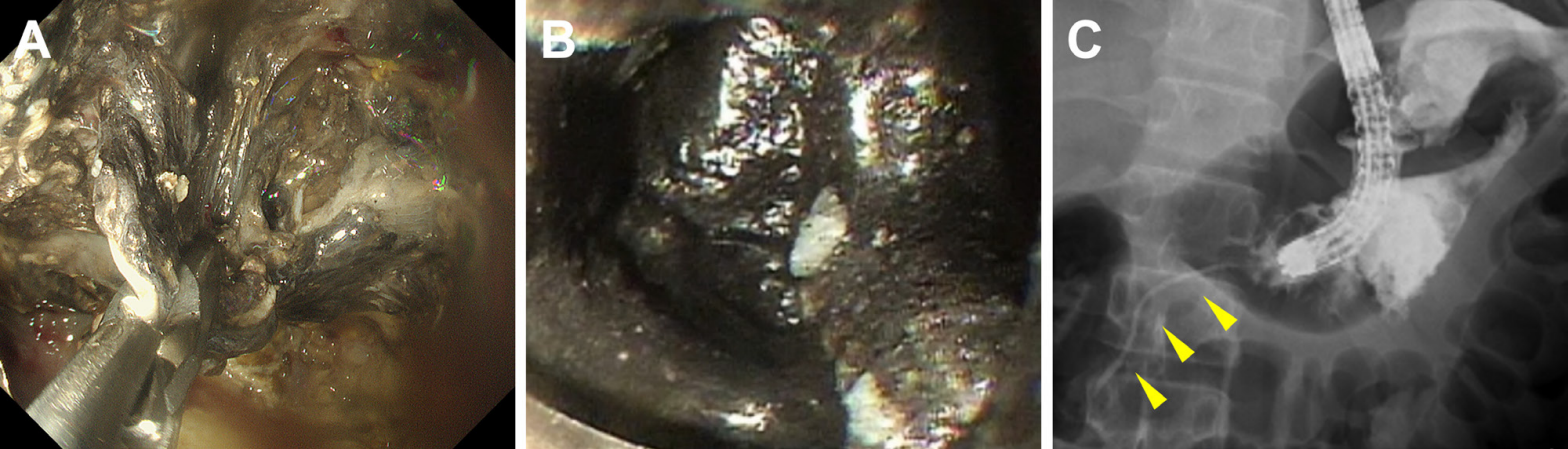
Endoscopic necrosectomy findings and confirmation of DPDS. (A) Endoscopic image showing necrosectomy using grasping forceps. (B) A deeper portion of the WON cavity revealing a large amount of retained solid necrotic debris despite multiple debridement sessions. (C) Contrast injection from within the cavity opacifying the main pancreatic duct, consistent with DPDS. DPDS, disconnected pancreatic duct syndrome; WON, walled-off necrosis.

The patient subsequently developed acute cholangitis caused by a common bile duct stone, despite the absence of gallstones on initial imaging [Figure 4A]. We hypothesized that repeated administration of fentanyl for pain control facilitated biliary stasis through opioid-induced sphincter of Oddi dysfunction, thereby promoting stone formation. In addition, increased intraductal pancreatic pressure likely contributed to ongoing DPDS, predisposing the patient to further hindering resolution of the WON.

**Figure 4.**
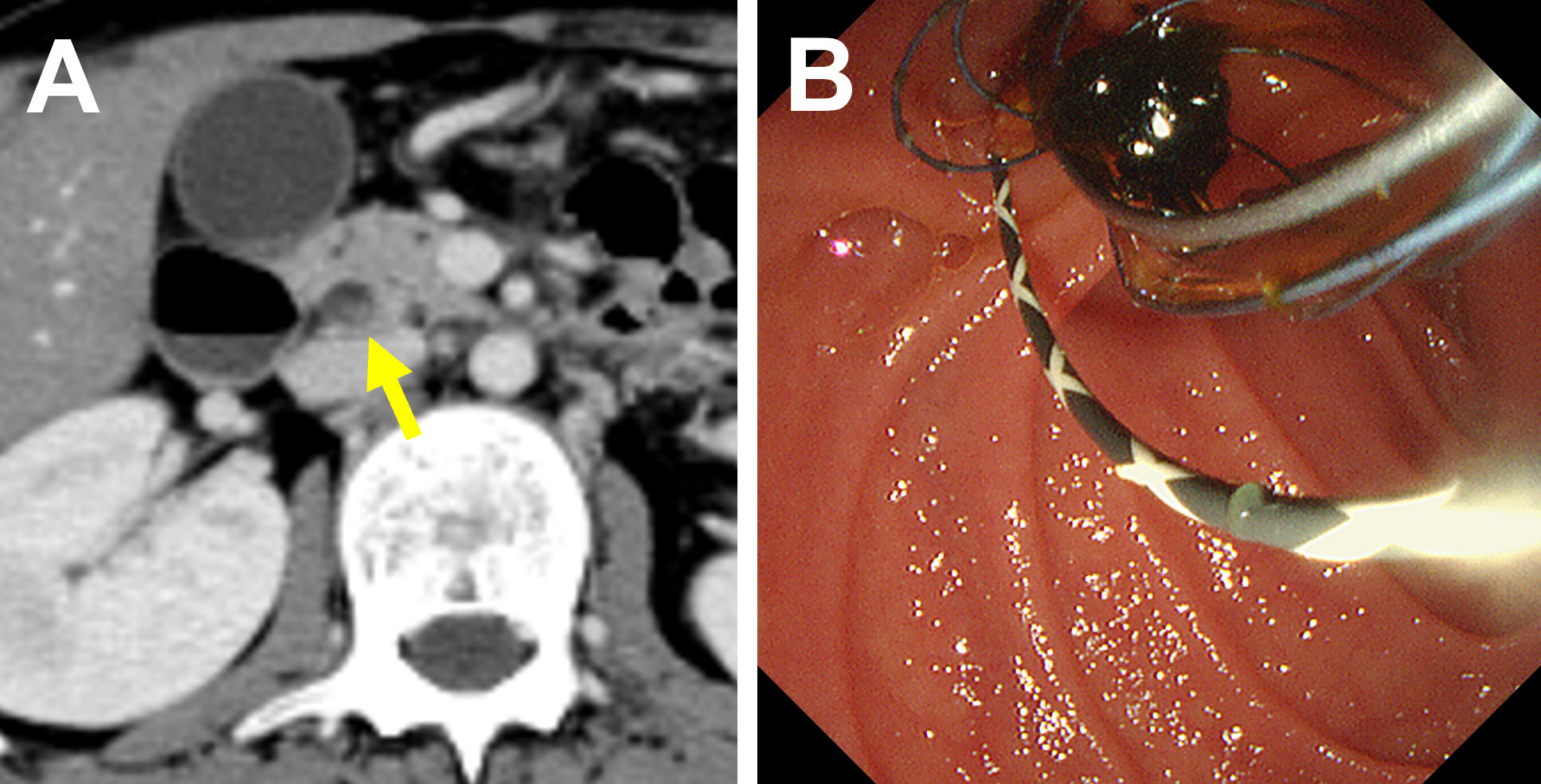
Development of choledocholithiasis during the clinical course. (A) CT image revealing a newly formed CBD stone. (B) Endoscopic image showing extraction of the CBD stone after endoscopic sphincterotomy. CBD, common bile duct; CT, computed tomography.

Therefore, endoscopic sphincterotomy and common bile duct stone extraction were performed [Figure 4B]. In addition, pancreatography demonstrated contrast leakage from the main pancreatic duct into the WON cavity [Figure 5A]; therefore, a transpapillary pancreatic duct stent (Geenen, Pancreatic Stent Sets 5Fr-3cm, Cook Medical, Bloomington, IN, USA) was placed to reduce ongoing pancreatic leakage [Figure 5B]. Follow-up computed tomography demonstrated a marked reduction of the WON without additional necrosectomy [Figure 6A, B]. A subsequent contrast study from within the cavity showed complete disappearance of the previously visualized ductal communication [Figure 6C]. The patient was discharged 2 weeks later and has had no recurrence of pancreatitis or WON.

**Figure 5.**
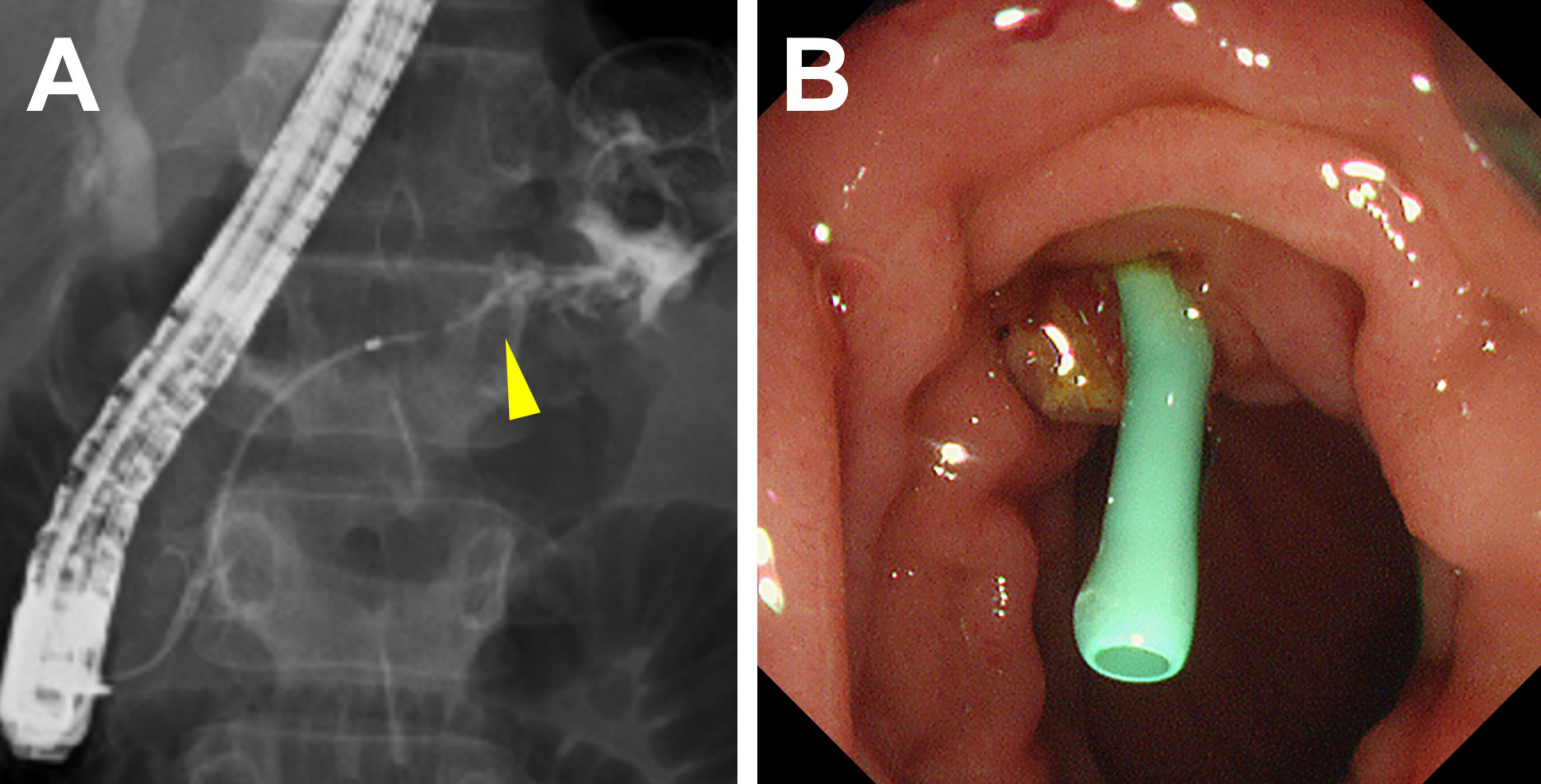
ERP demonstrating pancreatic duct disruption and decompression. (A) ERP image showing contrast leakage from the main pancreatic duct into the WON cavity. (B) Transpapillary pancreatic duct stent placed to reduce ongoing pancreatic leakage. ERP, endoscopic retrograde pancreatography; WON, walled-off necrosis.

**Figure 6.**
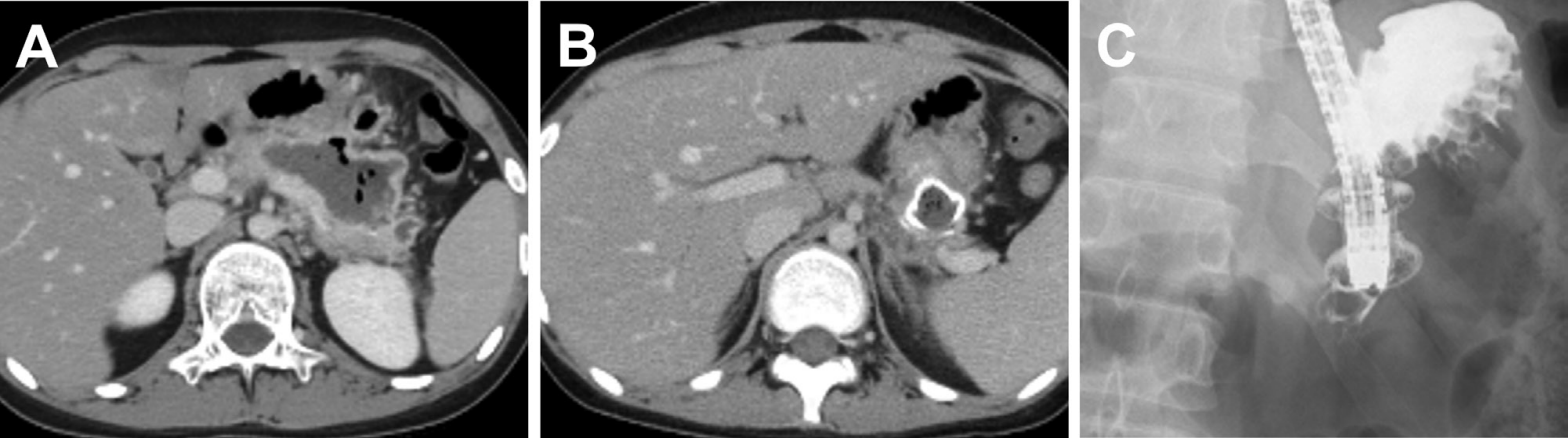
Resolution of the WON after pancreatic duct decompression. (A) CT before pancreatic duct stenting showing a persistent WON. (B) CT after stenting demonstrating marked shrinkage of the WON without further necrosectomy. (C) Subsequent contrast study showing complete disappearance of the communication between the WON and the main pancreatic duct, indicating resolution of DPDS. CT, computed tomography; DPDS, disconnected pancreatic duct syndrome; WON, walled-off necrosis.

This case illustrates that while EUS-guided lumen-apposing metal stent placement is highly effective for the management of WON, DPDS should be suspected in patients with refractory WON, and that pancreatic duct decompression may promote resolution of the collection.^[1–3]^ The striking change in imaging findings before and after ductal stenting provides a clear demonstration of the pathophysiologic impact of restoring pancreatic outflow.

## Supplementary Videos

Video 1: Contrast-based confirmation of disconnected pancreatic duct syndrome and its resolution after pancreatic duct decompression. Videos are only available at the official website of the journal (www.eusjournal.com).

## Ethical Statements

This study was conducted in accordance with the principles of the Declaration of Helsinki. Ethical approval was waived by the institutional review board due to the nature of a single case report. Written informed consent for publication of this case and accompanying images/video was obtained from the patient.

## Conflicts of Interest

The authors declare that they have no conflict of interest with regard to the content of this report.

## Author Contributions

M. Miyazawa contributed to the conception, procedure, data acquisition, and drafting of the manuscript. M. Yanagi and H. Takatori supervised the study and critically revised the manuscript. All authors approved the final version of the manuscript.

## Data Availability Statement

All relevant data are included in the article. Further inquiries can be directed to the corresponding author.
